# Improving treatment and liver fibrosis outcomes with metformin in HCV-HIV co-infected and HCV mono-infected patients with insulin resistance: study protocol for a randomized controlled trial

**DOI:** 10.1186/s13063-016-1454-6

**Published:** 2016-07-20

**Authors:** Mary-Anne Doyle, Joel Singer, Terry Lee, Miriam Muir, Curtis Cooper

**Affiliations:** Division of Endocrinology and Metabolism, Department of Medicine, University of Ottawa, Ottawa, ON Canada; Division of Infectious Diseases, Department of Medicine, University of Ottawa, Ottawa, ON Canada; Ottawa Hospital Research Institute, Ottawa, ON Canada; CIHR Canadian HIV Trials Network, Vancouver, BC Canada

**Keywords:** HCV, HIV, Liver fibrosis, Insulin resistance, Insulin sensitizer, Metformin, HCV antiviral therapy

## Abstract

**Background:**

Approximately 180 million people worldwide, (3 % of the world’s population) are infected with hepatitis C (HCV). Insulin resistance (IR) and type 2 diabetes (T2DM) are common extrahepatic manifestations of chronic HCV infection and associated with poor treatment and liver-related outcomes. The presence of these metabolic complications have been associated with poor response to interferon-based HCV antiviral therapy and increased risk of liver-related outcomes. Metformin, an insulin sensitizer is known to improve HCV treatment response and has been associated with a reduced risk of developing hepatocellular carcinoma (HCC). This study will evaluate the effect of metformin on preventing progression or promoting regression of liver fibrosis, rate of virologic cure (SVR) and other metabolic measures in HCV-HIV co-infected and HCV mono-infected study participants who have IR and are planning on initiating HCV treatment.

**Methods:**

This study is a prospective 48-week single-centre, randomized, open-label, controlled trial of HIV-HCV co-infected and HCV mono-infected patients with IR (HOMA-IR ≥ 2.0) who are planning to initiate HCV antiviral therapy. Sixty participants will be recruited from The Ottawa Hospital Viral Hepatitis Clinic. Participants will be randomized in a 1:1 ratio to either arm 1, metformin 2 g (1 g twice daily) plus lifestyle, or to arm 2, lifestyle alone. The primary outcome will be the change in FibroScan® score (kPa) from baseline to week 12 (start of HCV treatment), the end of HCV treatment (week 24) and 24 weeks post HCV treatment (week 48). Secondary outcomes include changes in liver fibrosis using AST to platelet ratio index, changes in glucose and lipid levels, anthropometric measures, changes in alpha-fetoprotein levels, patient acceptability, and changes in dietary and physical activity parameters.

**Discussion:**

This pilot study will be the first to evaluate the role of metformin on liver fibrosis in HCV-HIV co-infected and HCV mono-infected patients with IR receiving DAA HCV treatment. If metformin is effective in reducing liver fibrosis in this patient population, this will represent a well-tolerated, easy-to-administer, inexpensive therapy that will protect against negative HCV outcomes. This study will also be an opportunity to evaluate the impact of insulin resistance and hyperglycemia on viral clearance in HCV-infected patients treated with interferon-free regimens.

**Trial registration:**

ClinicalTrials.gov NCT02306070 version 4.0 (June 29, 2015)

**Electronic supplementary material:**

The online version of this article (doi:10.1186/s13063-016-1454-6) contains supplementary material, which is available to authorized users.

## Background

Approximately 180 million people worldwide (3 % of the world’s population) are infected with hepatitis C (HCV) [1]. Although HCV mainly affects the liver, the virus is also associated with several extrahepatic manifestations including hematologic, autoimmune, dermatologic, and endocrine disorders [2]. Insulin resistance (IR) and type 2 diabetes (T2DM) are the most common endocrine disorders in HCV-infected individuals. This association was first demonstrated in HCV-infected individuals with cirrhosis compared with individuals with cirrhosis from other causes (prevalence of T2DM 50 % versus 9 %, respectively) [3]. HCV is believed to directly impact insulin signaling by interacting with specific proteins such as serine/threonine kinases that subsequently inhibit insulin signaling molecules [4]. HCV is also believed to indirectly cause insulin resistance by inducing the production of pro-inflammatory cytokines that impair insulin signaling pathways in uninfected tissues [5].

The presence of IR and T2DM in HCV has been associated with poor HCV antiviral treatment response [6–8], acceleration of liver fibrosis [9, 10], increased risk for hepatocellular carcinoma (HCC) [11], higher transplant complication rates [12], and possibly increased morbidity from cardiovascular and metabolic complications [13]. At least one observational study identified improved IR in genotype 1 HCV-infected patients achieving viral clearance with HCV antiviral therapy [14].

Hepatic fibrosis is a response to liver injury that arises from the activation of hepatic stellate cells (HSCs) by inflammatory cytokines. Once HSCs are activated, they further release cytokines that promote inflammation, fibrosis, contraction and mitosis [15]. Insulin resistance is a major determinant of liver fibrosis, however, the mechanism by which IR promotes liver fibrosis is not well established.

In recent years, the treatment of HCV has evolved with the development of direct-acting antiviral (DAA) therapies (protease inhibitors, NS5a inhibitors, and nucleotide and non-nucleotide polymerase inhibitors). Compared to interferon and ribavarin therapies alone, these new treatments are associated with significantly improved sustained virological response (SVR) rates, shorter treatment duration and more favourable side effect profile.

The effect of DAA HCV treatments on insulin resistance and long-term risk of type 2 diabetes has yet to be clearly established. A randomized controlled trial of HCV mono-infected study participants receiving 14 days of monotherapy with the protease inhibitor danoprevir, found that serum HCV RNA and homeostatic model assessment of insulin resistance (HOMA-IR) correlated significantly (Spearman rho = 0.379, *p* < 0.0001) [16]. At the end of 14 days of danoprevir monotherapy the mean decrease in HCV RNA was 2.2 ± 1.3 log10 IU/ml (*p* < 0.0001) in patients who received the active drug (*n* = 40), which correlated with a decrease in mean HOMA-IR score by 1.6 ± 1.1 (*p* < 0.0001). In contrast, HCV-RNA and HOMA-IR remained unchanged in placebo recipients. The role of insulin resistance and hyperglycemia as predictors of SVR with DAA HCV treatment has not been clearly established. There are also no studies to date that have examined the role insulin resistance or hyperglycemia play in promoting progression or preventing regression of liver fibrosis in patients that have achieved SVR with HCV antiviral therapy.

In high-risk populations, metformin, an insulin sensitizer, has been shown to delay or prevent the onset of T2DM [17]. While it is unknown whether this specific benefit extends to those infected with HCV, metformin does improve HCV treatment and liver-related outcomes. At least one study showed treatment with metformin resulted in higher SVR rates when compared with the control group (59.2 % vs. 38.8 %, chi-square 4.083, *p* = 0.043) [18]. HCV-infected patients with cirrhosis and T2DM who were taking metformin had a decreased risk of developing HCC [19, 20].

At the cellular level, metformin is highly concentrated in the liver and improves IR through the activation of AMP-activated protein kinase (AMPK) which decreases gluconeogenesis in the liver and increases glucose uptake in the skeletal muscle [21]. In vitro studies have demonstrated that pharmacologic activation of AMPK with metformin may have antifibrotic effects by inhibiting the transforming growth factor beta (TGF-β1)-induced fibrogenic property of HSCs via transcriptional coactivator p300 [22]. TGF-β1 is the most characterized of the fibrotic cytokines and has been found to be increased in HCV-HIV co-infected patients [23]. It has also been proposed that the anticancer effects of metformin in HCV-infected patients may also be via AMPK and the inhibition of mammalian target of rapamycin (mTOR), which in turn regulates cell cycle progression and cell growth [24]. There are no studies to date that have looked at the effect of metformin in preventing progression of liver fibrosis or accelerating regression fibrosis in patients treated with HCV antiviral therapy.

Alpha-fetoprotein (AFP) is an oncofetal protein associated with hepatic malignancies and liver regeneration [25, 26]. HCV core protein, inflammation, necrosis and hepatocellular injury have all been suggested as causes for elevated AFP levels in chronic HCV infection [25–28]. Although type 2 diabetes and insulin resistance have been identified as risk factors for the progression of liver fibrosis and development of hepatocellular carcinoma the mechanism by which this occurs is not clear [9, 11, 29, 30]. The relationship between AFP and insulin resistance was recently examined in a retrospective analysis of 300 HCV-infected patients [31]. This study demonstrated that whole-body insulin resistance and hepatic fibrosis correlated directly with elevated levels of AFP lifestyle modification over a 3-month period correlated with improved insulin resistance and a reduction in AFP levels. This study draws attention to the need for further prospective studies to understand the relationship between insulin resistance, AFP, hepatic fibrosis and hepatocarcinogenesis. No studies have examined the effect of metformin, an insulin sensitizer, on AFP levels.

While SVR is associated with improved liver outcomes, the rate of liver fibrosis regression with SVR is variable and predictors of regression are not well established [32]. In addition, achieving SVR in patients with cirrhosis does not necessarily prevent decompensation or eliminate the risk of HCC. A better understanding of the role insulin resistance and impaired glucose metabolism have on these outcomes in HCV patients who achieve SVR are needed.

Identifying and targeting potentially modifiable risk factors such as IR may be of significant importance in preventing progression of and promoting regression of liver fibrosis, reducing mortality and improving outcomes for HCV-HIV co-infected and HCV mono-infected patients.

Given the above body of evidence and the established safety of metformin in HCV and HIV, we developed a protocol to evaluate the effect of metformin on preventing progression or promoting regression of liver fibrosis, rate of virologic cure (SVR) and other metabolic measures in HCV-HIV co-infected and HCV mono-infected study participants who have IR and are planning on initiating HCV treatment.

## Methods

### Hypothesis

The use of metformin will slow the progression and promote regression of liver fibrosis in HCV-HIV co-infected and HCV mono-infected patients with impaired insulin sensitivity (defined as HOMA-insulin resistance > 2.0).

### Objectives

The primary objective of this study will be to evaluate the role of metformin in preventing progression and promoting regression of liver fibrosis in HCV-HIV co-infected and HCV mono-infected participants with IR receiving antiviral HCV treatment as assessed by transient elastography (FibroScan®, Echosens, Paris, France).

As secondary objectives we will evaluate the effect metformin has on virologic response rates based on SVR (i.e. 12 weeks post HCV antiviral treatment and its effect on the progression and regression of liver fibrosis using aspartate aminotransferase (AST)-to-platelet ratio index (APRI). We will additionally consider the effect of metformin and lifestyle modification have on metabolic parameters (fasting insulin, fasting glucose and fasting lipid levels), inflammatory markers [interleukin 6 (IL-6), interleukin 8 (IL-8), tumor necrosis factor alpha (TNF-α), TGF-β, C-reactive protein (CRP], anthropometric measurements [weight, body mass index (BMI), waist circumference], AFP levels, HCV viral status, human immunodeficiency virus (HIV) viral status and liver enzymes. Patient acceptability of metformin will be evaluated in arm 1 using a patient acceptability questionnaire. All participants will receive lifestyle and dietary counselling at baseline and at 24-week intervals. The effects of lifestyle modification will be evaluated using dietary and physical activity questionnaires.

### Study design and setting

This study is a prospective 48-week single-centre, randomized, open-label, controlled trial of HIV-HCV co-infected and HCV mono-infected patients with IR (HOMA-IR ≥ 2.0) who are planning to initiate HCV antiviral therapy.

Sixty (60) participants meeting eligibility criteria will be randomized in a 1:1 ratio using variable block sizes to either arm 1, metformin 2 g [1 g twice a day (BID)] plus lifestyle, or to arm 2, lifestyle alone (the control arm). Randomization will be stratified according to HIV status and according to stage of liver fibrosis (F0–2 vs. F3–4) and will be conducted utilizing a web-based randomization system. The allocations will be generated by a statistician unassociated with the study using the SAS statistical program (SAS Institute, Inc., Cary, NC, USA) and uploaded into the randomization system. A log of all transactions including date and time of randomization, stratum and treatment allocation will be recorded. The randomization system will be accessed by the study coordinator at the study site when they have identified consenting eligible participants ready to be randomized.

**Fig. 1:**
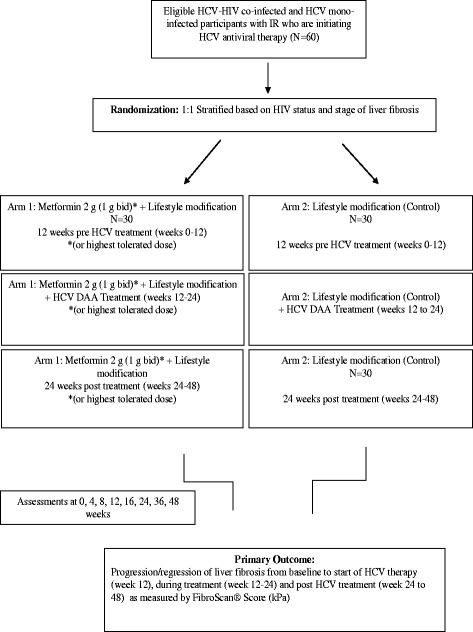
Flow diagram of study processes. Sixty participants meeting eligibility criteria will be randomized in a 1:1 ratio using variable block sizes to either arm 1, metformin 2 g (1 g BID) plus lifestyle (Metformin group), or to arm 2, lifestyle alone (Control group). *Abbreviations: HCV* hepatitis, *HIV* human immunodeficiency virus, *IR* insulin resistance*, BID* twice daily, *DAA* direct-acting antiviral therapy*, kPa* kilopascals

Arm 1 (Metformin group) will receive metformin and lifestyle treatment during a 12-week period prior to starting HCV therapy (week 0–12), during the 12-week treatment phase (week 12–24) and 24 weeks post HCV treatment (week 24–48). Participants who require > 12 weeks of HCV treatment will be excluded. If initiation of HCV treatment is delayed beyond 12 weeks, participants will continue on metformin during this time period and will receive less metformin post HCV treatment. Arm 2 (Control group) will receive lifestyle treatment alone during a 12-week period prior to starting HCV therapy (week 0–12), during the 12-week treatment phase (week 12–24) and 24 weeks post HCV treatment (week 24–48). A detailed study flow chart is depicted in Fig. 1.

### Selection of participants

Participants will be recruited from The Ottawa Hospital Viral Hepatitis Clinic. Study recruitment will begin in July 2016. All adult patients between the ages of 18 and 79 years of age, who have provided informed consent, have documented history of chronic HCV RNA infection with evidence of fibrosis on FibroScan® > 8.0 kilopascals (kPa) or liver biopsy score > 2 (Batts-Ludwig System [33] within 2 years, insulin resistance as determined by a HOMA-IR of > 2.0 at screening and intend to start an 8–12 week interferon (IFN)-free HCV antiviral therapy. If participants are HIV-infected and not on HIV antiviral therapy, they will require a CD4 count of at least 200 to be included in the study.

Individuals will be excluded from the study if they are: pregnant, suspected to be pregnant, planning to become pregnant or breastfeeding, have a chronic HBV infection, an HbA1c > 8.0 %, are using immune-suppressing medications, have an active malignancy, are currently or previously treated with metformin or other oral diabetes medications or insulin, have a history of pre-existing diabetes (type 1, type 2 or gestational diabetes), clinical evidence of decompensated cirrhosis (ascites, esophageal varices, hepatic encephalopathy, hepatocellular carcinoma), renal impairment [serum creatinine levels > = 136 umol/L (males), > = 124 umol/L (females)], history of congestive heart failure requiring pharmacologic therapy, Wilson’s disease, alpha-1 antitrypsin, hemochromatosis, biliary cirrhosis, alcohol consumption > 50 g/day on average, participation in other clinical investigations during the study, or a history of lactic acidosis irrespective of precipitating factors.

Active illicit drug use (IDU) and stable health illness will not be exclusionary assuming it is unlikely to compromise study adherence to protocol and study drug. In HIV-infected participants, HIV antiretroviral use and suppressed HIV viral load will not be required for participation.

### Informed consent and patient confidentiality

All participants will be given detailed oral and written information about the trial. Consent forms describing in detail the study medication/intervention(s), study procedures and risks will be given to each participant and written documentation of informed consent is required prior to starting study medication/intervention. Participants may withdraw consent at any time during the course of the trial.

All participant-related information including clinical records, laboratory specimens, evaluation forms, reports, will be kept strictly confidential. All records will be kept in a secure, locked location and only research staff will have access to the records. Participants will be identified only by means of a coded number specific to each participant and the site’s alphabetic letter code. All computerized databases will identify participants by numeric and alphabetic codes only, and will be password protected.

### Metformin dosing

Arm 1 participants will be provided with the following recommended dose adjustments and advised to increase the dose accordingly: week 1: 500 mg once daily (QD); week 2: 500 mg BID; week 3: 500 mg three times daily (TID); week 4: 1 g BID or 500 mg QD. Participants will also be encouraged to administer their study treatment with food whenever possible to minimize the gastric intolerances. If participants are unable to tolerate an increase in dose, they will continue on the last dose tolerated.

Metformin dosing will additionally be based on renal function and dosed as follows:

- if creatinine clearance (CrCl) 30–60 mL/min, metformin will be reduced to 50 % of the dose or 500 mg BID
- if CrCl < 30 mL/min, metformin will be stopped.

The potential side effects associated with metformin will be reviewed with each participant.

### Lifestyle modification intervention

Participants in both treatment arms will be given counselling on lifestyle and dietary modifications that may help to prevent progression of liver fibrosis. Participants will be given instructions on recommended types and duration of physical activity as per Canadian Diabetes Association practice guidelines. They will be encouraged to aim for 150 minutes of moderate to vigorous aerobic exercise and two to three sessions of resistance training each week. They will additionally be provided with tips on how to become more active.

Participants will further be provided with recommendations for dietary modifications and tips for healthy eating as provided by the Canadian Diabetes Association practice guidelines. Recommended limits for alcohol consumption will be reviewed. The educational sessions will be repeated during 6-month follow-up visits.

### Primary outcome

As the primary outcome we will evaluate the change in FibroScan® score (kPa) from baseline to week 12 (start of HCV treatment), the end of HCV treatment (week 24) and 24 weeks post HCV treatment (week 48) (Table 1).

**Table 1:** Schedule of events and data collection

Visit window			+/- 2 days						
Weeks	-4 (Screen)	0 (Baseline)	4	8	12	16	24	36	48
Informed consent	X								
Medical history	X								
Inclusion/exclusion criteria	X	X							
Drug, alcohol and smoking history	X								
Pregnancy test^a^	X	X^a^							
Medication review	X	X	X	X	X	X	X	X	X
FibroScan®^b^	X	X			X		X	X	X
Physical examination^c^ and vital signs	X	X^c^	X^c^	X^c^	X^c^	X^c^	X^c^	X^c^	X^c^
Hepatitis B serology^d^	X								
HCV RNA viral load^e^	X^e^	X	X	X	X	X	X	X	X
HCV genotype^d^		X							
HIV viral load^d,e^ (as per SOC)	X^e^	X			X		X	X	X
CD4 count^e^ (as per SOC)	X^5^	X			X		X	X	X
Hematology^f^ and chemistry^g^	X	X	X	X	X	X	X	X	X
HbA1c	X	X			X		X	X	X
Fasting insulin and glucose	X	X	X	X	X	X	X	X	X
Fasting lipids^h^		X			X		X	X	X
Inflammatory markers/research blood		X	X	X	X	X	X	X	X
TSH		X				X	X		X
AFP levels		X			X		X	X	X
2-hr OGTT		X							X
Adverse events			X	X	X	X	X	X	X
Anthropometric measures^i^	X^i^	X			X		X	X	X
Counselling on lifestyle modification		X					X		X
Block food frequency questionnaire		X					X		X
International physical activity questionnaire		X					X		X
Audit-C and illicit drug use questionnaires		X			X		X	X	X
TSQM for patient acceptability (arm 1)				X			X		X
Study drug accountability/dispensation (arm 1)		X	X	X	X		X	X	X
Birth control review	X	X	X	X	X		X	X	X

*HCV* hepatitis C, *HIV* human immunodeficiency virus, *HbA1c* glycated hemoglobin, *TSH* thyroid-stimulating hormone, *AFP* alpha-fetoprotein, *OGTT* oral glucose tolerance test, *TSQM* Treatment Satisfaction Questionnaire for Medication

^a^If randomization occurs > = 4 weeks from last pregnancy test, perform pregnancy test at baseline

^b^FibroScan® can be done at screening if necessary for inclusion; baseline test can be done on different day than rest of visit if necessary (within 4 weeks of baseline). FibroScan® window can be +/− 7 days of the week 12 visit, +/− 21 days of week 24 and 36 visits, and up to 21 days before the week 48 visit

^c^Targeted physical exam: vitals, cardiac, respiratory and abdominal exams; exam as pertinent to patient complaints

^d^Tests to be done if not already in participant medical records [hepatitis B (HBV) needs to be within 6 months of screening visit; HIV needs to be within 1 month of screening]

^e^If results available within 1 month of screening visit, no need to retest

^f^Hematology: complete blood count (CBC) with differential, platelets, international normalized ratio (INR)

^g^Chemistry: electrolytes, aspartate aminotransferase (AST), alanine aminotransferase (ALT), gamma-glutamyl transferase (GGT), lipase, albumin, direct bilirubin, lactate, creatinine, estimated glomerular filtration rate (eGFR)

^h^Lipids: total cholesterol, high-density lipoprotein (HDL), low-density lipoprotein (LDL), triglycerides

^i^Anthropometric measures: height, weight, body mass index (BMI), waist circumference, hip circumference, waist-to-hip ratio. At screening, only height and weight documented

### Secondary outcomes

The following will be evaluated as secondary outcomes: (i) virological response rates (SVR 12 weeks post HCV antiviral therapy) will be compared between treatment groups; (ii) the change in APRI measurements from baseline to start of HCV treatment, to the end of HCV treatment (week 24) and 24 weeks post HCV treatment (week 48) will be compared between treatment groups; (iii) changes in glucose metabolism (HOMA-IR, fasting insulin, glucose levels) from baseline to 4, 8, 12, 24, 36 and 48 weeks; (iv) changes in lipid levels [total cholesterol, low-density lipoprotein cholesterol (LDL-c), high-density lipoprotein cholesterol (HDL-c), triglycerides] from baseline to 0, 12, and 36 and 48 weeks; (v) changes in anthropometric measures (waist circumference, body weight and BMI) from baseline to 0, 4, 8, 12, 24, 36 and 48 weeks; (vi) changes in liver-related inflammatory markers by (IL-6, IL-8, TNF-α, TGF-β, C-reactive protein (CRP)) from baseline to 0, 4, 8, 12, 24 and 36 weeks; (vii) changes in AFP levels from baseline to 0, 12, 24, 36 and 48 weeks; (viii) participant acceptability to study medication dosing will be assessed (in arm 1 only) at weeks 8, 24, and 48 weeks by questionnaire. Changes in scores from week 8 to end of study will be evaluated; (ix) changes in diet and physical exercise parameters from baseline to 24 and 48 weeks using dietary and physical activity questionnaires (Table 1).

### Safety assessments

Metformin has been studied in both HCV and HIV mono-infected patients with no increase in adverse events (AE) [18, 34]. Metformin does have the potential risk of lactic acidosis. A reduced dose (50 % of maximal dose) is used for patients with a CrCl < 60 mL/min. It is not recommended for use in patients with a creatinine clearance of < 30 mL/min. Patients will be cautioned against excessive alcohol intake, either acute or chronic when taking metformin, since alcohol intake potentiates the effect of metformin on lactate metabolism [35, 36].

A Data and Safety Monitoring Committee (DSMC) will be implemented to safeguard participant safety. All members will be independent from the study investigators. This committee will conduct regularly scheduled meetings to review the study progress including recruitment and conduct, and will identify any potential safety signals in the study population.

At each contact with the participant, information regarding adverse events (AEs) will be elicited by appropriate questioning and examinations. Any AE that occurs between the time that the participant is randomized and the time that s/he departs the study at the end of the final follow-up visit (or at the time of early withdrawal of the participant from the study for any reason) is to be recorded. Participants will also be monitored during the 48-week study period for serious adverse events (SAEs). If an SAE is ongoing at the time a participant discontinues/completes the trial, the SAE will be followed until the investigator agrees that the event is satisfactorily resolved, becomes chronic, or that no further follow-up is required (Table 1). 

### Study treatment adherence

The following criteria will be used to define compliance during this study:

1. Adherence with study treatment will be assessed based on participant-provided information and tablet count, i.e., drug accountability, during follow-up visits. Non-adherence will be considered for participants who have an average daily intake of less than 80 % of their maximum tolerated dose.
2. Attend baseline visit and all follow-up visits.
3. Adherence with HIV antiretroviral therapy (ART) (if applicable) and HCV antiviral therapy will be documented as standard of care.

### Sample size calculation

To date there have been no studies that have investigated the benefits of improved IR in the HIV-HCV co-infected population or in HCV mono-infected patients. Studies that have investigated the effects of HCV antiviral therapy on FibroScan® score reported a 32 % reduction in mean score (10.6 +/- 4.8 kPa) compared with baseline in patients that obtained a treatment response [37]. Based on these study results, a sample size of 24 participants in each group would provide approximately 80 % power to detect a 4 kPa difference between the two treatment arms assuming a similar standard deviation. We anticipate a 20 % loss to follow-up, so an additional six participants will be randomized per group to account for loss to follow-up and to allow secondary outcome analyses. Using regression of the final measurement on the initial measurement will likely improve the power relative to using simple difference scores.

All analyses will be based on intention-to-treat basis and per protocol analysis. Intention-to-treat population will be defined as the set of all patients randomized.

### Statistical analysis

As the primary outcome of this study, the influence of metformin on preventing and promoting regression of liver fibrosis in HCV antiviral treatment recipients will be assessed by transient elastography. The change in transient elastography scores (kPa) at week 48 (24 weeks post HCV therapy) between groups will be assessed using linear regression adjusted for baseline score. We will also compare the trend on fibrosis progression over the course of the study (12, 24, 36 and 48 weeks) between groups using mixed-effects regression analysis. All analyses will be based on intention-to-treat basis. A sensitivity analysis of the change at 48 weeks will be conducted using multiple imputations to include those participants who do not have 48-week follow-up scores.

The same set of analyses based on a per protocol basis will also be performed. For the analysis, which examines the outcome longitudinally, participants who terminated the assigned treatment prematurely will be censored at the time that they went off treatment.

Secondary outcome measures include: virological response rates, changes from baseline to week 48 in indirect measures of liver fibrosis (APRI), inflammatory markers, metabolic measures, anthropometric measurements, HCV and HIV viral loads, AFP levels and liver function. Linear regression adjusted for baseline score will be used to compare the outcome at week 48 between groups. Mixed-effects regression analysis will also be used to compare the trend over time between groups.

To account for potential confounding factors (i.e., HCV/HIV viral status, alcohol consumption, IR, BMI) associated with changes in liver fibrosis, a sensitivity analysis based on multivariate regression analysis will be performed.

Patient acceptability of daily long-term study treatment use will be evaluated (in arm 1 only) using the Treatment Satisfaction Questionnaire for Medication. Patient acceptability will be assessed based on change in median score from week 8 (first administration of questionnaire) to week 24 and week 48. Descriptive statistics will be used to summarize the data.

Baseline characteristics will be expressed as mean and standard deviation (SD) for continuous variables and percentages for categorical variables. Baseline variables to be considered include: age, gender, ethnicity, HCV genotype and viral load, HIV viral load and CD4 count (as per SOC), HbA1c, HOMA-IR, FibroScan® scores, smoking history, alcohol consumption, and illicit drug use.

Lifestyle modification will be evaluated through changes in physical activity (changes in median MET/week, percentage change in participants classified as low, medium and high level of activity) and dietary changes (change in median daily caloric intake, daily consumption of fat, protein, carbohydrates and micro/macro nutrients). Linear regression adjusted for baseline score will be used to compare the outcome at week 48 between groups. Mixed-effects regression analysis will also be used to compare the trend over time between groups.

Data will be collected on liver-related complications (progression of liver fibrosis stage, HCC, transplantation) and mortality. We anticipate that the event rate will be low given the small sample size and short duration of follow-up.

Subgroup analyses will be conducted based on SVR to compare the effect virological response influences liver fibrosis and the benefit of metformin in reducing progression of fibrosis. Subgroup analyses will also be conducted based on HIV status to compare the influence of HIV infection on progression of liver fibrosis and benefit of metformin in reducing the progression of fibrosis.

Subgroup analyses based on metformin doses (full dose versus not) in the metformin group and weight loss in patients in both treatment arms will also be conducted. These additional analyses will be useful in determining if lower doses can achieve the same benefit and the impact that potential weight loss associated with this medication may have on steatosis and improving liver outcomes respectively.

Subgroup analyses will also be conducted based on end of study HOMA-IR score (<2 vs. > 2) to evaluate if the benefits of metformin are dependent or independent of the insulin-sensitizing effects of this treatment.

As a sensitivity analysis, we will also consider using multiple imputation techniques to impute missing data for the primary outcome at 48 weeks. Linear regression adjusted for baseline score will then be used to compare the groups.

When half (n = 30) of the participants have completed their 48-week visit, an interim analysis of efficacy will be conducted. The same technique and analytic strategy will be used to analyze the primary end point, transient elastography score (kPa) at 48 weeks. An intention-to-treat approach will be taken, linear regression adjusting for baseline score, and missing imputations to account for missing data will be used. The O’Brien-Fleming approach using the Lan-DeMets alpha spending function will be used, so that the criterion for statistical significance at the first analysis will be 0.005 and the criterion at the final analysis will be 0.048 to maintain 80 % power overall. This analysis will be supplemented by a mixed-effects regression model examining the outcome at all time points for all patients available, and adjusting for baseline values.

### Early termination

The criteria for premature discontinuation of further study medication for an individual participant in arm 1 are as follows: treatment-related toxicity, requirement for prohibited concomitant medications, clinical reasons believed to be life-threatening by the physician, even if not addressed in the toxicity section of the protocol.

The participant will continue to be followed with the participant’s permission if the study medication is prematurely discontinued. If the participant discontinues study medication between scheduled visits, the investigator may request that the participant visit the clinic as soon as possible for lab and safety assessments and to return the study medication for accountability purposes. There will be no changes to the follow-up visit schedule, except no study medication will be administered. If the participant chooses not to remain in the study, then the participant will be withdrawn from the study and participant will be asked to come in for an early termination visit.

The criteria for permanent withdrawal from the study for an individual participant are as follows:

loss to follow-up, pregnancy or suspected pregnancy, HCV treatment required for >12 weeks, request of the participant to withdraw from the trial, any clinical AE, laboratory abnormality, intercurrent illness, other medical condition or situation occurs such that continued participation in the study would not be in the best interest of the participant, the participant is judged by the investigator to be at significant risk of failing to comply with the provisions of the protocol as to cause harm to self or seriously interfere with the validity of the trial results, progression of diabetes requiring additional diabetes medication (i.e. oral diabetes medications or insulin therapy).

In the event that the participant is withdrawn from the study due to an AE, this must be recorded on the case report form (CRF). The subject should be followed and treated by the investigator until the abnormal parameter or symptom has resolved or stabilized.

### Trial registration and dissemination

This study has been registered with ClinicalTrials.gov (Identifier Number NCT02306070, https://clinicaltrials.gov/ct2/show/NCT02306070). A SPIRIT checklist is provided as an additional file (see Additional file 1). The findings of this trial will be submitted to a peer-reviewed journal and abstracts will be presented at relevant national and international conferences.

## Discussion

HCV antiviral therapy has evolved rapidly in recent years and access to these medications has improved. While SVR is associated with improved liver outcomes, the rate of liver fibrosis regression with SVR is variable and predictors of regression are not well established [32]. In addition, achieving SVR in patients with cirrhosis does not necessarily prevent decompensation or eliminate the risk of HCC. A better understanding of the role insulin resistance and impaired glucose metabolism have on these outcomes in HCV patients who achieve SVR are needed.

Identifying and targeting potentially modifiable risk factors such as IR and T2DM may be of significant importance in preventing progression of and promoting regression of liver fibrosis, reducing mortality and improving outcomes for HCV-HIV co-infected and HCV mono-infected patients.

This pilot study will be the first to evaluate the role of metformin on liver fibrosis in HCV-HIV co-infected and HCV mono-infected patients with IR receiving DAA HCV treatment. If metformin is effective in reducing liver fibrosis in this patient population, this will represent a well-tolerated, easy-to-administer, inexpensive therapy that will protect against negative HCV outcomes. This study will also be an opportunity to evaluate the impact of insulin resistance and hyperglycemia on viral clearance in HCV-infected patients treated with interferon-free regimens. In addition, the study will further explore the relationship between HCV, insulin resistance and AFP levels. This knowledge will inform patient care, translate into improved therapeutic outcomes for liver and metabolic diseases, and guide further innovative research in this area.

## Trial status

Recruitment for this study will begin in July 2016.

## Abbreviations

AE, adverse event; AFP, alpha-fetoprotein; AMPK, AMP-activated protein kinase; APRI, AST to platelet ratio index; ART, antiretroviral therapy; BID, twice daily; BMI, body mass index; CBC, complete blood count; CrCl, creatinine clearance; CRF, case report form; CRP, C-reactive protein; CTN, Canadian HIV Trials Network; DAA, direct-acting antivirals; DSMC, Data and Safety Monitoring Committee; eGFR, estimated glomerular filtration rate; HBV, hepatitis B; HCC, hepatocellular carcinoma; HCV, hepatitis C; HDL-c, high density lipoprotein cholesterol; HIV, human immunodeficiency virus; HOMA-IR, homeostatic model assessment of insulin resistance; HSC, hepatic stellate cells; ICH, International Conference on Harmonization; IDU, illicit drug use; IFN, interferon; IL-6, interleukin 6; IL-8, interleukin 8; INR, international normalized ratio; IR, insulin resistance; IRB/REB, institutional review board; kPa, kilopascal; LDL-c, low-density lipoprotein cholesterol; mTOR, mammalian target of rapamycin; N, number; OGTT, oral glucose tolerance test; PI, principal investigator; QD, once daily; REB, research ethics board; SAE, serious adverse event; SOP, standard operating procedure; SVR, sustained virological response; T2DM, type 2 diabetes; TGF-β, transforming growth factor beta; TID, three times daily; TNF-α, tumour necrosis factor alpha; WHO, World Health Organization

## Additional file

Additional file 1: SPIRIT 2013 checklist: recommended items to address in a clinical trial protocol and related documents. (DOC 121 kb)
